# Supplementary material for: Genome-Wide Association Study of Diabetogenic Adipose Morphology in the GENetics of Adipocyte Lipolysis (GENiAL) Cohort
Source: Cells. 2020 Apr 27;9(5):1085. doi: 10.3390/cells9051085 (PMC7291295; doi:10.3390/cells9051085)
Supplement: Supplementary file 1 [file cells-09-01085-s001.pdf]

Supplementary Materials

Table 1. SNPs associated with adipose morphology with *P* < 10-5.

	POS	ID	REF	ALT	A1	BETA	SE	L95	U95	P	A1_FREQ	MACH_R2
1	3297459	rs201839757	I	D	I	198	43	113	283	5.75E-06	0.0126	0.57
1	48004858	rs12032932	T	C	T	167	36	96	238	4.40E-06	0.0110	0.95
1	76545413	rs10443175	T	C	T	-40	9	-57	-22	8.15E-06	0.2247	0.97
1	157782519	rs77346326	T	C	T	136	28	81	191	1.28E-06	0.0203	0.88
2	19739010	rs3914966	T	C	T	-34	7	-48	-19	4.64E-06	0.4256	1.02
2	19739976	rs1427547	C	T	T	-33	7	-47	-19	7.49E-06	0.4281	1.02
2	38470091	rs74470768	G	T	G	172	39	96	247	9.40E-06	0.0100	0.90
2	38486755	rs57981885	T	C	T	163	36	91	234	9.25E-06	0.0105	0.96
2	38491982	rs17022448	T	C	T	163	36	92	235	8.49E-06	0.0106	0.95
2	38507228	rs17022498	C	G	C	156	35	88	225	8.83E-06	0.0115	0.95
2	38507905	rs75560317	G	C	G	157	35	89	226	8.28E-06	0.0114	0.95
2	38508250	rs147711728	T	C	T	164	36	93	234	6.30E-06	0.0108	0.95
2	170990253	rs185528286	C	T	C	159	34	91	226	4.45E-06	0.0153	0.74
3	189409341	rs7618742	T	C	T	48	10	28	69	4.94E-06	0.1560	0.92
3	189410000	rs2378515	A	G	G	54	10	34	73	5.29E-08	0.1802	0.94
3	189410345	rs2889919	C	T	T	53	10	34	72	8.68E-08	0.1778	0.94
3	189410351	rs4274726	A	T	T	53	10	34	72	8.66E-08	0.1779	0.94
3	189411881	rs9840152	G	C	C	52	10	33	71	1.67E-07	0.1751	0.95
3	189415174	rs73195987	C	T	C	47	10	26	67	8.55E-06	0.1532	0.94
3	189423435	rs75613372	T	A	T	48	10	27	68	5.67E-06	0.1525	0.95
3	189426360	rs73195998	C	T	C	47	10	27	67	6.74E-06	0.1528	0.96
4	170028965	rs202156267	A	C	A	-164	33	-229	-99	9.12E-07	0.0313	0.40
6	97632917	rs148707864	G	T	G	140	31	79	200	7.01E-06	0.0148	0.95
6	130537399	rs11965136	A	G	A	99	21	57	140	4.19E-06	0.0319	0.95
6	130538552	rs76542576	A	T	A	96	21	55	137	6.07E-06	0.0328	0.95
6	130539859	rs75893354	A	G	A	99	21	57	140	4.17E-06	0.0318	0.95

6	130539934	rs143814468	D	I	D	98	21	56	140	5.99E-06	0.0331	0.91
6	130540268	rs117998221	A	G	A	99	21	57	140	4.17E-06	0.0318	0.95
6	130541689	rs75623854	C	T	C	98	21	57	140	3.68E-06	0.0323	0.95
6	130570534	rs62431212	T	G	T	100	21	59	141	1.96E-06	0.0334	0.94
6	130581980	rs62431222	A	G	A	102	21	61	144	1.36E-06	0.0329	0.94
7	52698748	rs150804725	I	D	I	-112	24	-159	-64	4.65E-06	0.0297	0.78
7	156412814	rs849073	G	C	G	-34	8	-48	-19	9.16E-06	0.4351	0.95
8	1784216	rs111719741	G	A	A	-33	7	-47	-18	9.96E-06	0.4146	1.02
8	1784219	rs111406201	C	T	T	-33	7	-47	-18	9.96E-06	0.4146	1.02
8	1784343	rs11984532	T	C	C	-33	7	-47	-18	9.97E-06	0.4143	1.02
8	1784364	rs11988258	G	A	A	-34	7	-48	-20	4.06E-06	0.4201	1.02
8	32613177	rs75468268	G	C	G	155	35	87	223	8.95E-06	0.0134	0.83
8	69224040	rs814465	T	C	C	82	17	49	114	1.04E-06	0.0537	0.94
8	69224115	rs10622380	I	D	D	77	17	44	109	3.96E-06	0.0581	0.89
8	141147356	rs142956251	A	G	A	155	35	87	224	8.43E-06	0.0139	0.80
9	21219185	9:21219185_A_G	G	A	G	161	34	95	227	2.08E-06	0.0128	0.92
9	21256050	rs151152540	C	T	C	158	33	93	223	2.22E-06	0.0130	0.93
9	21279590	rs146702051	T	C	T	157	33	92	221	2.12E-06	0.0129	0.96
9	21340235	rs145072648	C	G	C	163	34	97	230	1.59E-06	0.0126	0.93
9	21348642	rs146632290	A	C	A	156	33	92	220	2.22E-06	0.0132	0.95
9	21350904	rs138085325	T	C	T	163	34	97	230	1.88E-06	0.0124	0.92
9	21354605	rs144317189	T	A	T	160	34	94	227	2.60E-06	0.0128	0.91
9	21355471	rs144198601	G	A	G	161	34	95	227	2.19E-06	0.0129	0.92
9	21382372	rs141316837	G	A	G	154	33	89	219	3.38E-06	0.0134	0.92
9	30859764	rs138591003	A	C	A	-178	39	-255	-100	7.76E-06	0.0108	0.84
10	97277536	rs189712177	G	C	G	186	40	107	265	4.43E-06	0.0115	0.71
10	128606691	rs71490795	A	G	A	121	27	69	174	6.52E-06	0.0225	0.84
11	23509085	rs148697259	A	G	A	133	30	75	192	9.22E-06	0.0181	0.83
11	99369450	rs72991567	G	C	G	67	14	39	95	2.54E-06	0.0804	0.89

12	73510344	rs60148685	D	I	D	34	8	19	48	8.09E-06	0.4437	0.95
13	30552356	rs11301895	D	I	I	-72	16	-104	-40	9.47E-06	0.0617	0.87
13	30571045	rs200421910	D	I	I	-64	14	-92	-36	8.33E-06	0.0988	0.73
13	109383269	rs151235076	T	C	T	188	41	108	267	4.27E-06	0.0097	0.83
16	2390565	rs45501400	T	G	T	169	38	94	243	9.89E-06	0.0163	0.57
17	22020568	rs201766885	C	T	C	113	24	65	161	4.24E-06	0.0464	0.50
17	22020584	rs201968922	C	T	C	112	24	64	160	5.71E-06	0.0468	0.49
17	22020615	rs74393147	C	T	C	163	36	92	234	7.33E-06	0.0229	0.45
19	53280252	rs10414169	T	A	T	131	29	74	189	8.82E-06	0.0183	0.87
22	32558640	rs144964180	A	G	A	160	35	90	229	7.28E-06	0.0122	0.87
22	49801423	rs9627723	T	C	C	45	10	26	64	4.15E-06	0.3654	0.60

Onlys results for SNPs with MAF > 1% are shown. I = insertion; D = deletion; A1 = effect allele

A

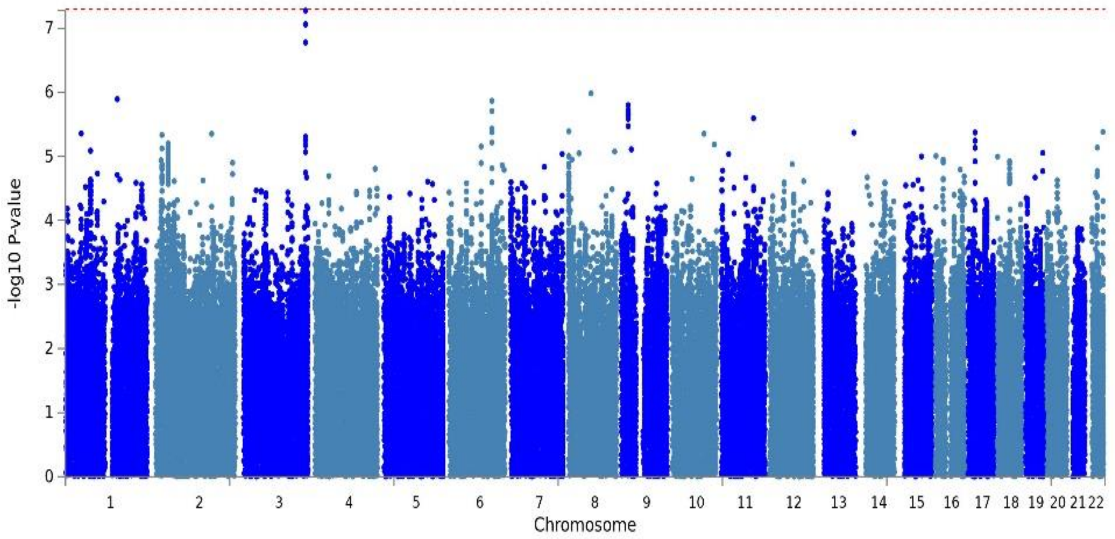

B

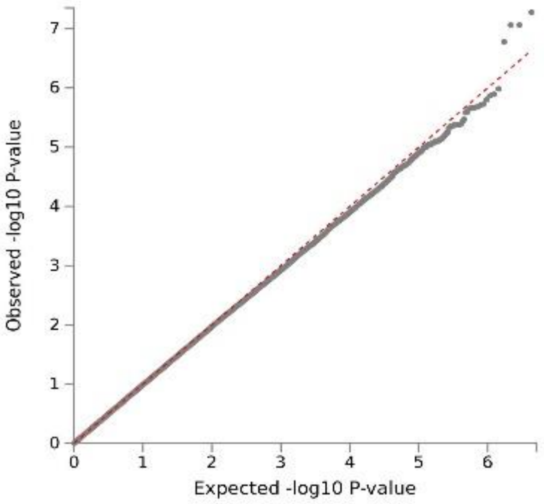

Figure S1: QQ and Manhattan plots for A) Adipose morphology. **B**) QQ plot, the red dotted line indicates the null distribution. In the Manhattan plot, the horizontal red line represents the threshold for GWAS significance ($p < 5 \times 10^{-8}$).

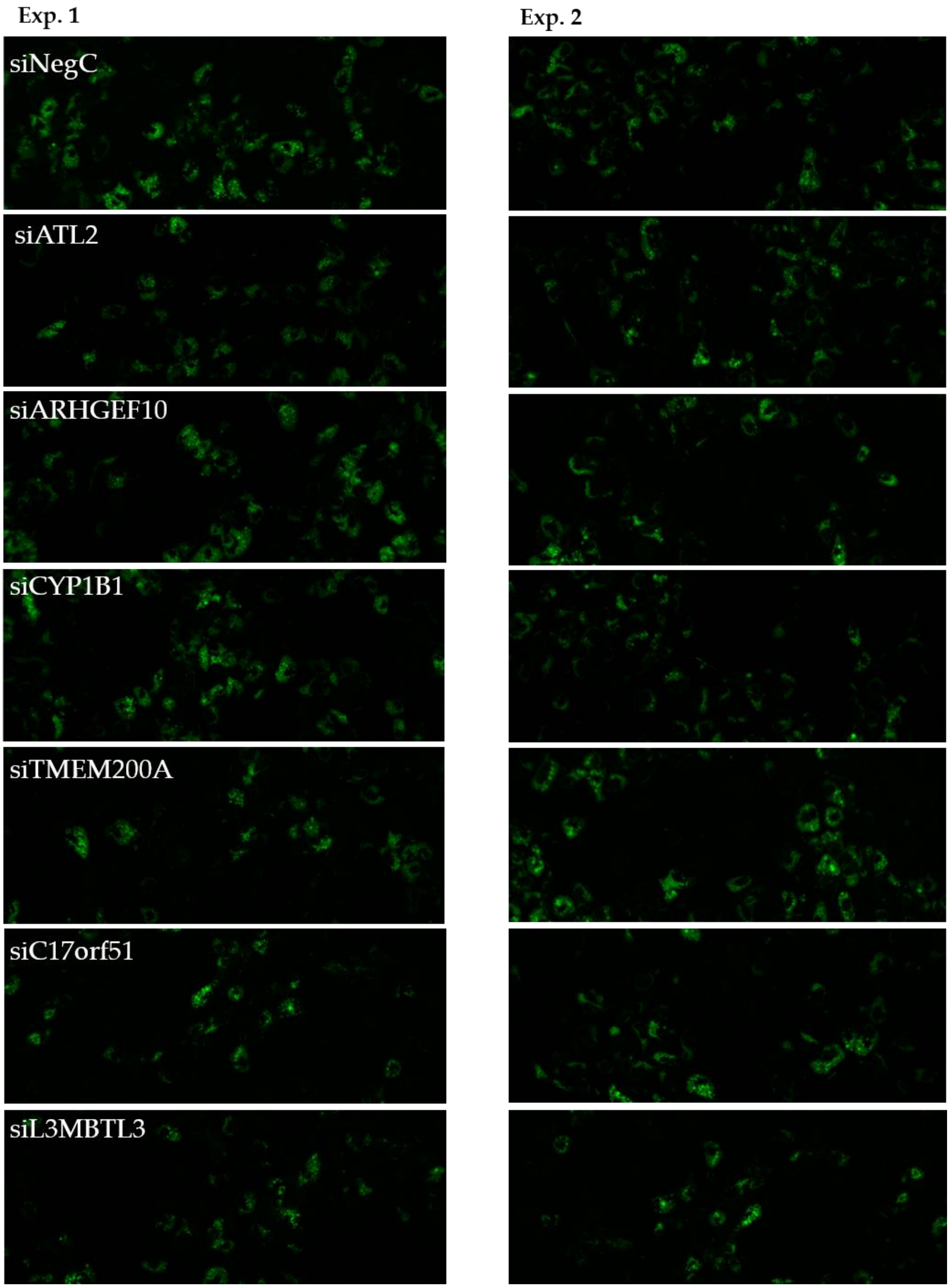

Figure S2. Images of neutral lipids stained by Bodipy (493/503 nM) in hMSCs at day 9 of differentiation in vitro that were transfected with siRNA against ATL2, AHRGEF10, CYP1B1, TMEM200A, C17orf51,L3MBTL3 and NegC. Images are two independent experiments.
